# Supplementary material for: Comparison of Hemodynamic and Cerebral Oxygenation Responses during Exercise between Normal-Weight and Overweight Men
Source: Healthcare (Basel). 2023 Mar 22;11(6):923. doi: 10.3390/healthcare11060923 (PMC10048205; doi:10.3390/healthcare11060923)
Supplement: Supplementary file 1 [file healthcare-11-00923-s001.zip › healthcare-2162413-supplementary.pdf]

(Supplement)

Table S1. Cerebral oxygenation responses during 30-min continuous exercise and post-exercise period.

	NW(n=18)	OW (n=14)	P
ΔO_2Hb (μmol)			
warm	6.4±6.4	5.4±6.8	0.781
Exercise10 min	22.7±9.6	19.9±11.7	0.628
Exercise 20 min	27.2±9.5	22.7±13.9	0.481
Exercise 30 min	27.6±10.8	20.0±18.1	0.346
Cool down	24.5±8.2	17.1±19.5	0.351
Post10 min	19.7±7.0	5.8±16.4	0.055
Post20 min	13.9±7.0	1.0±13.1	0.039*
Post30 min	12.3±8.7	0.60±10.9	0.049*
ΔHHb (μmol)			
warm	0.12±1.6	-0.23±1.5	0.689
Exercise10 min	2.1±2.9	2.4±2.0	0.829
Exercise 20 min	5.0±2.8	2.6±3.3	0.176
Exercise 30 min	5.6±2.4	0.95±5.9	0.065
Cool down	4.4±2.5	0.19±7.4	0.158
Post10 min	3.3±4.0	-3.4±7.7	0.063
Post20 min	2.1±3.6	-3.3±6.3	0.069
Post30 min	2.1±2.8	-3.0±5.4	0.046*

* : $p < 0.05$, between group comparison

Table S2. Hemodynamics responses during 30-min continuous exercise and post-exercise period.

	NW(n=18)	OW (n=14)	P
Stroke volume(ml/beat)			
Rest	72.8±3.6	84.8±4.2	0.009*
Rest	90.6±3.4	96.8±4.1	0.160
Warm up	104.3±4.0	107.7±4.7	0.375
Exercise10 min	108.1±4.0	110.4±4.8	0.661
Exercise 20 min	112.1±4.2	115.9±4.9	0.681
Exercise 30 min	92.4±4.1	97.6±4.1	0.404
Cool down	76.2±2.8	82.3±3.3	0.352
Post10 min	74.2±2.8	81.1±3.3	0.292
Post20 min	72.1±2.4	79.6±2.9	0.098
Cardiac output(L)			
Rest	5.4±0.37	7.7±0.45	0.009*
Warm up	9.6±0.50	10.8±0.60	0.160
Exercise10 min	15.0±0.70	15.6±0.83	0.375
Exercise 20 min	16.2±0.78	16.8±0.92	0.661
Exercise 30 min	16.9±0.85	17.6±1.0	0.681
Cool down	10.9±0.77	12.4±0.91	0404
Post10 min	7.1±0.44	8.1±0.51	0.352
Post20 min	6.3±0.39	7.6±0.46	0.392
Post30 min	5.8±0.35	7.3±0.42	0.098
SVRi (d.s.cm^{-5} /m)	NW	OW	p
Rest	2395±485	2140±604	0.007*
Warm up	1339±258	1489±291	0.717
Exercise10 min	843±138	1050±259	0.238
Exercise 20 min	800±150	965±238	0.331
Exercise 30 min	752±125	922±222	0.176
Cool down	1217±414	1330±330	0.795
Post10 min	1801±358	2030±352	0.564
Post20 min	2006±321	2158±475	0.702
Post30 min	2193±386	2205±475	0.145

* : $p < 0.05$, between group comparison
